# Acetate Metabolism in Archaea: Characterization of an Acetate Transporter and of Enzymes Involved in Acetate Activation and Gluconeogenesis in *Haloferax volcanii*

**DOI:** 10.3389/fmicb.2020.604926

**Published:** 2020-12-04

**Authors:** Tom Kuprat, Ulrike Johnsen, Marius Ortjohann, Peter Schönheit

**Affiliations:** Institut für Allgemeine Mikrobiologie, Christian-Albrechts-Universität, Kiel, Germany

**Keywords:** *Haloferax volcanii*, acetate transporter ActP, archaea, AMP-forming acetyl-CoA synthetase, malic enzyme, PEP synthetase

## Abstract

The haloarchaeon *Haloferax volcanii* grows on acetate as sole carbon and energy source. The genes and proteins involved in uptake and activation of acetate and in gluconeogenesis were identified and analyzed by characterization of enzymes and by growth experiments with the respective deletion mutants. (i) An acetate transporter of the sodium: solute-symporter family (SSF) was characterized by kinetic analyses of acetate uptake into *H. volcanii* cells. The functional involvement of the transporter was proven with a Δ*ssf* mutant. (ii) Four paralogous AMP-forming acetyl-CoA synthetases that belong to different phylogenetic clades were shown to be functionally involved in acetate activation. (iii) The essential involvement of the glyoxylate cycle as an anaplerotic sequence was concluded from growth experiments with an isocitrate lyase knock-out mutant excluding the operation of the methylaspartate cycle reported for *Haloarcula* species. (iv) Enzymes involved in phosphoenolpyruvate synthesis from acetate, namely two malic enzymes and a phosphoenolpyruvate synthetase, were identified and characterized. Phylogenetic analyses of haloarchaeal malic enzymes indicate a separate evolutionary line distinct from other archaeal homologs. The exclusive function of phosphoenolpyruvate synthetase in gluconeogenesis was proven by the respective knock-out mutant. Together, this is a comprehensive study of acetate metabolism in archaea.

## Introduction

Acetate serves as substrate for catabolism and anabolism of several aerobic and anaerobic bacteria, eukarya and archaea. The metabolism of acetate in bacteria and eukarya is well studied; it involves uptake of acetate into the cells followed by activation of acetate to acetyl-CoA, the common intermediate for both catabolism and anabolism. It has been assumed that acetate uptake across the cytoplasmic membrane predominantly proceeds by passive diffusion of the undissociated acid (pKs 4.78). However, at neutral and alkaline pH acetic acid is almost completely present in the dissociated anionic form suggesting the operation of a transport system for acetate. So far, acetate transporters have been reported only in few bacteria, e.g., *E. coli* and *Corynebacterium glutamicum* (Gimenez et al., 2003; Jolkver et al., 2009), and in eukarya, e.g., *Saccharomyces cerevisiae* and *Candida* species (Alves et al., 2020) and recently in methanoarchaea, in *Methanosarcina* species (Welte et al., 2014; Ribas et al., 2019).

The mechanism of acetate activation to acetyl-CoA has been studied in detail for many bacteria and eukarya. The most common enzyme of acetate activation is the AMP-forming acetyl-CoA synthetase (ACS) that catalyzes the ATP- and CoA-dependent activation of acetate generating acetyl-CoA, AMP and pyrophosphate (acetate + ATP + CoA → acetyl-CoA + AMP + PP_i_) representing a high affinity mechanism of acetate activation (Brown et al., 1977; Starai and Escalante-Semerena, 2004; Wolfe, 2005). Several bacteria, e.g., *C. glutamicum* and *Azotobacter vinelandii*, activate acetate to acetyl-CoA via a low affinity system involving two enzymes, acetate kinase (AK) (acetate + ATP → acetylphosphate + ADP) and phosphotransacetylase (PTA) (acetylphosphate + CoA → acetyl-CoA + phosphate) (Tauchert et al., 1990; Reinscheid et al., 1999). Few bacteria, e.g., *E. coli*, were shown to activate acetate either by ACS or by AK and PTA depending on the acetate concentration (Kumari et al., 2000; Wolfe, 2005). An alternative mechanism of acetate activation involving succinyl-CoA: acetate CoA transferases has been reported for few sulfate reducers and the eukaryotic protist *Trichomonas vaginalis* (Thauer et al., 1989; van Grinsven et al., 2008). In the domain of archaea acetate activation has been reported for the aerobic halophilic archaea *Haloarcula marismortui* and *Haloferax volcanii* and for anaerobic methanogens, e.g., *Methanosarcina* and *Methanosaeta* species (Thauer et al., 1989; Bräsen and Schönheit, 2001, 2005). *Methanosaeta* species, *M. consilii* and *M. thermophila*, activate acetate via ACS (Ingram-Smith and Smith, 2007; Berger et al., 2012) whereas acetate activation in *Methanosarcina* species involves the AK-PTA mechanism (Thauer et al., 1989). In haloarchaea, which do not contain genes encoding AK and PTA, the activation of acetate has been proposed to proceed via ACS. An ACS from *H. marismortui* has been characterized (Bräsen and Schönheit, 2005).

Following the activation of acetate, acetyl-CoA serves as intermediate of both catabolism and anabolism. In most aerobic organisms acetyl-CoA is metabolized in the catabolism to CO_2_ involving enzymes of the citric acid cycle and respiratory chain. In anaerobic methanogens acetyl-CoA is converted to CH_4_ and CO_2_, generating ATP. In the anabolism of most aerobic bacteria and eukarya the glyoxylate cycle is operative as anaplerotic sequence by converting two molecules acetyl-CoA to malate. The generation of the gluconeogenetic intermediate phosphoenolpyruvate (PEP) from malate proceeds in most organisms via oxaloacetate by PEP carboxykinase; an alternative route has been reported for few bacteria that involves malic enzyme and PEP synthetase (Sauer and Eikmanns, 2005). In the domain of archaea, evidence for the operation of a glyoxylate cycle has been reported for *H. volcanii* and *Sulfolobus acidocaldarius* and the key enzymes isocitrate lyase and malate synthase have been characterized (Serrano et al., 1998; Serrano and Bonete, 2001; Uhrigshardt et al., 2002). So far, an essential involvement of the glyoxylate pathway in these archaea, as concluded from growth experiments with deletion mutants of key enzymes, have not yet been reported. This is important to exclude the operation of alternative anaplerotic pathways such as the methylaspartate cycle recently described in the haloarchaeon *Haloarcula marismortui* (Borjian et al., 2016). Further, the enzymes involved in PEP synthesis from malate have not been reported in haloarchaea so far.

Here we report the identification and characterization of genes and enzymes involved in the uptake and activation of acetate and in gluconeogenesis. We identified an acetate transporter, four paralogous AMP-forming acetyl-CoA synthetases that are essential for acetate activation, and describe enzymes that catalyze the formation of PEP from acetyl-CoA.

## Materials and Methods

### Growth of *H. volcanii* H26 and Deletion Mutants

*Haloferax volcanii* H26 and deletion mutants were grown aerobically at 42°C in synthetic medium with uracil (50 μg/ml) and 15 mM glucose or 40 mM acetate (Sutter et al., 2016). For complementation experiments, deletion mutants were transformed with plasmids carrying the respective genes under the control of a tryptophanase promoter; growth was performed in medium without uracil and expression of the target gene was induced by the addition of tryptophan (80 μM). Growth was followed by measuring the optical density at 578 nm over time using the photometer Ultrospec 2000 (GE Healthcare). The concentration of acetate was determined enzymatically.

### Generation of Deletion Mutants

Deletion mutants were generated by using the pop-in/pop-out strategy (Allers and Mevarech, 2005; Reinhardt et al., 2019). Flanking regions of the target genes were amplified and fused by PCR. These PCR products were each ligated into pTA131, and the resulting plasmids were multiplied in *Escherichia coli* XL1 Blue MRF’ cells. *H. volcanii* H26 was transformed with the respective plasmids (pop-in). Pop-in clones were selected in uracil-free synthetic medium with 1% casamino acids and each pop-in clone was passaged for several times. For pop-out selection, cultures were streaked onto agar plates containing uracil (30 μg/ml) and 5-fluoroorotic acid (50 μg/ml) followed by passaging in liquid medium. The deletion mutants were identified by PCR and verified by Southern hybridization. Double, triple and quadruple mutants were generated successively using the respective *H. volcanii* mutant.

### Acetate Transport Experiments

Acetate-grown *H. volcanii* H26 cells from the exponential growth phase were centrifuged for 20 min at 8000 x g and 4°C. Cells were washed and concentrated in 100 mM Tris–HCl, pH 8.0, containing 139 mM KCl, 2.1 M NaCl, to an optical density at 578 nm of 50. Aliquots of 80 μl were taken and transport experiments were started by the addition of 20 μl radiolabeled [1–^14^C]-acetate solution (109 μM, 0.01 μCi/probe) followed by incubation at room temperature for 20–300 s. Acetate uptake was stopped by the addition of 1 ml ice-cold buffer (see above) and samples were directly transferred on a vacuum filtration unit fitted with a Whatman GF/C filter membrane. Filters were immediately washed with 7 ml ice-cold buffer (see above), transferred to scintillation vials containing 5 ml of scintillation fluid (Quicksafe A, Zinsser Analytic) and analyzed using a Packard TriCarb liquid scintillation counter (PerkinElmer Inc., Waltham, MA, United States).

### Purification of ACS2 From *H. volcanii*

Acetate-grown cells were suspended in buffer A (0.1 M Tris–HCl, pH 8.0, 2 M ammonium sulfate, 50 mM MgCl_2_) and were disrupted using a French pressure cell followed by centrifugation. The supernatant was applied on a Phenyl-Sepharose column (2.6 × 10 cm; GE Healthcare) and elution of protein was performed with a decreasing gradient of ammonium sulfate. Fractions containing ACS activity were adjusted to an ammonium sulfate concentration of 1.5 M and were applied on a HiTrap Phenyl-Sepharose column (1 ml; GE Healthcare), that was equilibrated in buffer B (50 mM Tris–HCl, 1.5 M ammonium sulfate, pH 7.5). Elution was performed with a decreasing gradient of ammonium sulfate in buffer B. Fractions containing ACS activity were applied to a Superdex 200 HiLoad gel filtration column (1.6 × 60 cm) and elution of protein was performed in buffer C (50 mM Tris–HCl, 1.5 M KCl, pH 7.5). Fractions containing ACS activity were diluted with buffer D (50 mM Tris–HCl, 2 M ammonium sulfate, pH 8) and applied to a HiTrap Butyl-Sepharose column (1 ml; GE Healthcare). Protein was eluted with a decreasing gradient of ammonium sulfate. At this stage, protein was apparently pure as checked by SDS-PAGE. The encoding gene of purified enzyme was identified by MALDI-TOF MS analysis.

### Homologous Overexpression and Purification of Recombinant Enzymes

Target genes were amplified from genomic DNA of *H. volcanii*. DNA fragments were each ligated into the plasmid pTA963 followed by transformation of *H. volcanii* H1209. The expression of target genes in *H. volcanii* H1209 was induced by the addition of 2 mM L-tryptophan, followed by further growth for 16 h at 42°C (Allers et al., 2010; Reinhardt et al., 2019). *H. volcanii* H1209 cell pellets were suspended in 50 mM Tris–HCl, pH 8.2, containing 1.5 M KCl and 5 mM imidazole, and disruption of cells was performed by passing through a French pressure cell followed by a centrifugation step. The supernatants were applied on a nickel-nitrilotriacetic acid (Ni-NTA) column (GE Healthcare), and elution of recombinant proteins was performed with 100 or 150 mM imidazole. Recombinant proteins were further purified using a Superdex 200 column in 50 mM Tris–HCl, pH 7.5, containing 1.5 M KCl. To prevent loss of PPS activity 5 mM DTE was added to each buffer. Protein concentration was determined by the Bradford method with bovine serum albumin as standard and purity of the enzymes was analyzed by SDS-PAGE.

### Determination of Native Molecular Masses of Enzymes

Size exclusion chromatography was carried out with a flow rate of 1 ml per min on a Superdex 200 HiLoad column (1.6 × 60 cm). Calibration of the column was performed with the HMW and LMW kits (GE Healthcare) as specified by the manufacturer.

### Enzyme Assays

Enzyme activities were measured at 42°C. V_max_- and K_m_-values were calculated with the Origin2015 software using the hyperbolic function according to the Michaelis-Menten equation.

ACS activity (acetate + CoA + ATP → acetyl-CoA + AMP + PP_i_) was measured both in the direction of acetyl-CoA formation (A) and in the direction of acetate formation (B) according to Bräsen and Schönheit (2005). (A) The CoA- and acetate-dependent AMP formation from ATP was measured by coupling the reaction with the oxidation of NADH via pyruvate kinase, lactate dehydrogenase and adenylate kinase. The assay mixture contained 2.5 mM MgCl_2_, 0.3 mM NADH, 1 mM PEP, 2-5 mM ATP, 10 mM acetate, 3 mM CoA, 9.3 U adenylate kinase, 4 U pyruvate kinase and 6 U lactate dehydrogenase in 100 mM Tris–HCl, pH 7.0, 1 M KCl (ACS7 and ACS1), 100 mM Tris–HCl, pH 7.5, 1.5 M KCl (ACS9) or in 100 mM Tris–HCl, pH 7.5, 2 M KCl (ACS2). (B) The PP_i_- and AMP-dependent CoA release from acetyl-CoA was monitored with Ellman’s thiol reagent (5,5-dithio-bis-(2-nitrobenzoic acid), DTNB) by measuring the formation of thiophenolate anion at 412 nm (ε_412_ = 13.6 mM^–1^ cm^–1^). The assay mixture contained 2.5 mM MgCl_2_, 0.1 mM DTNB, 2 mM acetyl-CoA, 2 mM AMP, 1 mM PP_i_ and ACS in 100 mM Tris–HCl, pH 7.0, 1 M KCl (ACS7) or in 100 mM Bis Tris–HCl, pH 7.0, 2 M KCl (ACS1).

Malic enzyme activities (malate + NAD(P)^+^ → pyruvate + NAD(P)H + CO_2_) were measured in the direction of pyruvate formation (A) and in the direction of malate formation (B). The oxidation/reduction of pyridine nucleotides were monitored at 340 nm (ε_340_ = 6.2 mM^–1^ cm^–1^). For Mae1 the assay mixture (A) contained 0.1 M BisTris, 4 M KCl, pH 5.75, 5 mM MnCl_2_, 0.5 mM NADP^+^, 1.5 mM L-malate and malic enzyme and (B) contained 0.1 M BisTris, 4 M KCl, pH 5.75, 5 mM MnCl_2_, 0.2 mM NADPH, 10 mM NaHCO_3_, 20 mM pyruvate and malic enzyme. NADH or NAD^+^ as cofactors were tested by replacing NADPH or NADP^+^ at equal concentrations. Assay B was used to determine the pH optimum and the optimal KCl and MnCl_2_ concentration. For Mae2 activity the assay mixture (A) contained 0.1 M BisTris, pH 7.0, 1.5 M KCl, 5 mM MnCl_2_, 0.5 mM NAD^+^, 10 mM L-malate. NADP^+^ as cofactor was tested at concentrations of up to 0.5 mM. The assay mixture (B) contained 100 mM BisTris, pH 6.0, 1.5 M KCl, 0.3 mM NADH, 5 mM MnCl_2_, 30 mM NaHCO_3_ and pyruvate up to 70 mM.

PEP synthetase activity (pyruvate + ATP → PEP + AMP + P_i_) was measured in the direction of PEP formation using a discontinuous assay (Eyzaguirre et al., 1982). The assay mixture contained 100 mM Tris–HCl, pH 8.0, 2 M KCl, 20 mM MgCl_2_, 25 mM 1,4-dithioerythritol (DTE), 1 mM pyruvate and enzyme. The reaction was started by the addition of 5 mM ATP and was incubated at 42°C up to 20 min. Aliquots (200 μl) were taken and mixed with 800 μl buffer (100 mM Tris–HCl, pH 8.0, 1 mM ADP, 20 mM MgCl_2_, 0.4 mM NADH) and 9 U lactate dehydrogenase. The amount of PEP formed was determined by the oxidation of NADH after addition of 6 U pyruvate kinase which convert PEP to pyruvate. PEP synthetase activity was analyzed in the direction of pyruvate formation by a continuous assay. The assay mixture contained 0.1 M Tris–HCl, pH 8.0, 2 M KCl, 20 mM MgCl_2_, 25 mM DTE, 0.3 mM NADH, 1 mM PEP, 10 mM KH_2_PO_4_, 5 mM AMP, 9 U lactate dehydrogenase and enzyme. The amount of pyruvate formed was determined by the oxidation of NADH.

### Transcriptional Analysis

RNA from exponentially grown cells of *H. volcanii* H26 was isolated as described previously (Johnsen et al., 2013). Northern blot analysis was carried out with 15 μg RNA as described previously (Pickl et al., 2012). The probe against *ssf* was generated by PCR using the PCR digoxigenin (DIG) probe synthesis kit (Roche Diagnostics).

## Results and Discussion

*Haloferax volcanii* grew on 40 mM acetate with a doubling time of 12.6 hours up to an optical density at 578 nm of 1.7 (Supplementary Figure S1). Acetate was completely consumed during the exponential growth phase. In the following, we report the identification and characterization of an acetate transporter and of enzymes involved in acetate activation and in gluconeogenesis.

### Acetate Uptake in *H. volcanii* Is Mediated by a Symporter of the SSF Family

**FIGURE 1:**
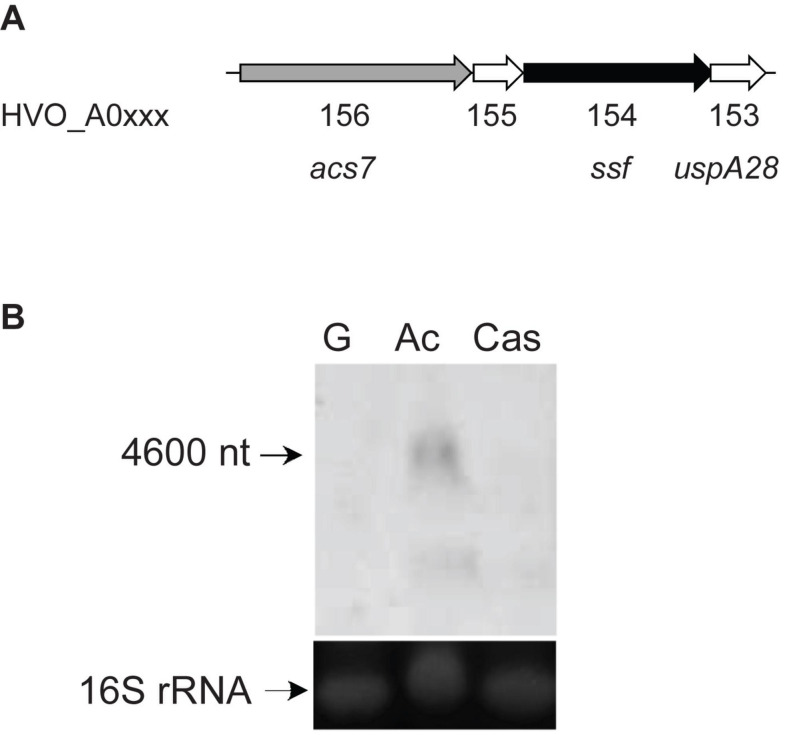
Genes involved in acetate uptake and activation in *H. volcanii*. **(A)** Genome organization of *acs7*, HVO_A0155, *ssf*, and *uspA28* encoding an acetyl-CoA synthetase (ACS7), a hypothetical protein, a putative acetate transporter (ActP) and an universal stress protein (UspA), respectively. **(B)** Northern blotting was performed with a probe against *ssf* using RNA from cells grown on acetate (Ac), glucose (G) and casamino acids (Cas). 16S rRNA was used as a loading control.

In the genome of *H. volcanii* a gene cluster was identified that comprises four genes (*ssf*, *acs7*, *uspA28*, and HVO_A0155) encoding a putative acetate-transporter designated ActP (acetate transport protein) of the sodium: solute symporter family (SSF), a putative AMP-forming acetyl-CoA synthetase (ACS7), a putative universal stress protein (UspA) and a hypothetical protein (Figure 1A). Transcription of the *ssf* gene was analyzed in acetate-, glucose- and casamino acids-grown cells by a Northern blot experiment. The analysis revealed a specific signal at about 4600 nucleotides in acetate-grown cells that corresponds to the sum of the four genes *acs7*, HVO_A0155, *ssf* and *uspA* (4625 nucleotides) indicating co-transcription of the genes (Figure 1B). No transcript could be detected in cells grown on glucose and casamino acids indicating an acetate-specific induction of the operon and thus an involvement of ActP and ACS7 in transport and activation of acetate, respectively.

**FIGURE 2:**
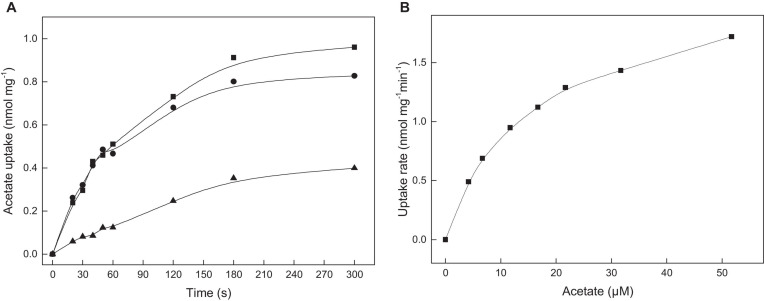
ActP mediated acetate uptake in cell suspensions of *H. volcanii*. **(A)** Acetate-grown cells of *H. volcanii* wild type (•), of the Δ*ssf* mutant (▲) and the mutant complemented with *ssf in trans* (■). **(B)** Acetate uptake rate in dependence of the acetate concentration measured in wild type cells.

To prove the functional involvement of ActP in acetate uptake, transport assays with ^14^C-labeled acetate were performed with acetate-grown cells of *H. volcanii*. As shown in Figure 2A, wild type cells catalyzed the uptake of acetate that was proportional up to 60 seconds with an acetate uptake rate of about 0.6 nmol mg^–1^ min^–1^. A rate-dependence of acetate uptake on the acetate concentration revealed a saturation kinetic indicating that acetate is taken up by a carrier mediated mechanism rather than by diffusion. Half-maximal saturation of uptake was found at 13.7 μM acetate (Figure 2B). To attribute the uptake of acetate to the putative transporter ActP, a *ssf* deletion mutant was generated. Growth of the Δ*ssf* mutant on acetate, both at 40 mM and 8 mM, was not affected as compared to the wild type; however, the acetate uptake rate of the Δ*ssf* mutant was reduced by 80% to 0.15 nmol mg^–1^ min^–1^ (Figure 2A). The wild type uptake rate could be completely restored by complementation of Δ*ssf* with the *ssf*-gene *in trans*. Taken together the data indicate that acetate uptake in *H. volcanii* is mediated by a transporter of the SSF family. The observed unrestricted growth of *H. volcanii* on high acetate concentrations (40 mM or 8 mM) might be due to passive diffusion of acetate and/or to the activity of an alternative transporter.

### Sequence Comparison of ActP From *H. volcanii* and Phylogenetic Affiliation

**FIGURE 3:**
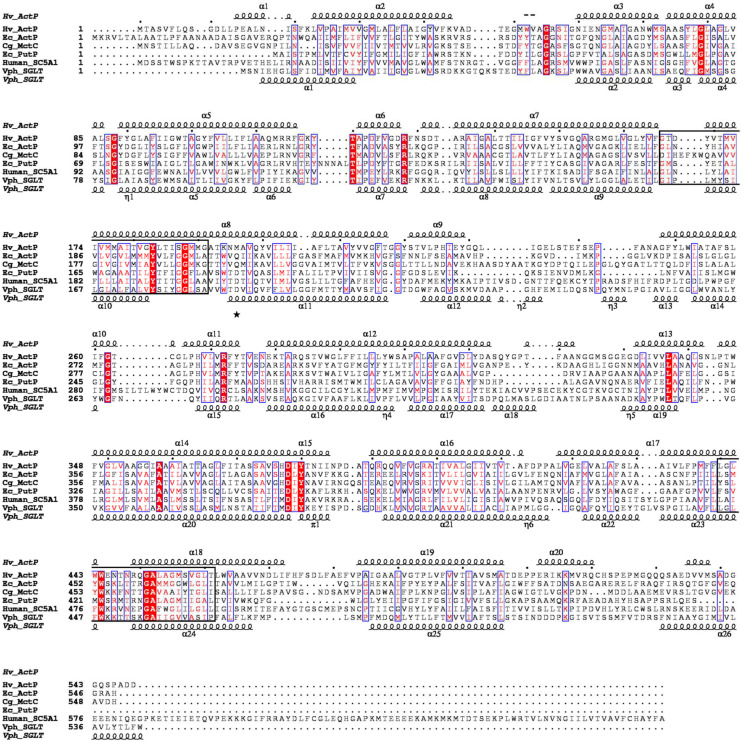
Multiple amino acid sequence alignment of ActP from *H. volcanii*, of proton: monocarboxylic acid symporters (MctC) and of sodium: proline (PutP) and sodium: sugar symporter (SGLT) of the SSF family. The alignment was calculated with ClustalX (Larkin et al., 2007). Shading in red indicates degree of sequence conservation. Structural-based secondary structure elements of SGLT from *V. parahaemolyticus* and the predicted secondary structure (Jones, 1999) of ActP from *H. volcanii* are displayed using ESPript 3.0 (Robert and Gouet, 2014). The position of conserved aspartate residues proposed to be essential for sodium dependency is marked by an asterisk. The signatures I and II of sodium: solute symporter family are indicated by boxes (Prosite entry PDOC00429). Abbreviations: Hv, *Haloferax vocanii*; Ec, *E. coli*; Cg, *Corynebacterium glutamicum*; Vph, *Vibrio parahaemolyticus*.

The acetate transporter ActP from *H. volcanii* shows high sequence identity to proteins of the sodium: solute symporter family, SSF, which belongs to the APC (amino acid-polyamine-organo cation) superfamily of secondary carriers (Vastermark et al., 2014). The SSF family comprises several subfamilies including, e.g., proton: monocarboxylate symporters and sodium coupled symporters for proline and sugars (Quick et al., 2001; Hosie et al., 2002; Jung, 2002; Gimenez et al., 2003; Faham et al., 2008; Jolkver et al., 2009; Jung et al., 2012). A multiple-sequence alignment of ActP from *H. volcanii* and selected characterized members of the SSF family from bacteria and eukarya is shown in Figure 3. The alignment includes the acetate transporter ActP from *E. coli* and the monocarboxylate transporter MctC from *C. glutamicum*, the sodium: proline symporter PutP from *E. coli*, the sodium: glucose symporter SLC5A1 from *Homo sapiens* and the crystallized sodium: galactose symporter SGLT from *Vibrio parahaemolyticus*. These transporters show a sequence identity to the *H. volcanii* ActP of 23–30%. All sequences contain the two consensus motifs typical for the SSF family (Prosite database PDOC00429). The predicted secondary structure of ActP from *H. volcanii* matches well to the secondary elements of the SGLT structure from *Vibrio parahaemolyticus* (Figure 3; Faham et al., 2008). The sequence comparison also shows that the sodium-dependent transport systems, i.e., PutP of *E. coli* and the sugar transporter from human and *V. parahaemolyticus*, contain each a conserved aspartate residue which has been proposed to be essential for the sodium-dependent transport systems (Quick and Jung, 1998; Quick et al., 2001; Faham et al., 2008). At the equivalent position ActP from *H. volcanii* and *E. coli* and MctC from *C. glutamicum* contain instead a glutamine or methionine (Figure 3). In accordance, ActP from *E. coli* and MctC from *C. glutamicum* have been shown to function as proton-dependent symporter (Gimenez et al., 2003; Jolkver et al., 2009). Also, ActP from *H. volcanii* likely functions as proton-coupled acetate-transporter since acetate uptake was inhibited by 50% upon addition of 10 μM of the protonophore CCCP (not shown).

**FIGURE 4:**
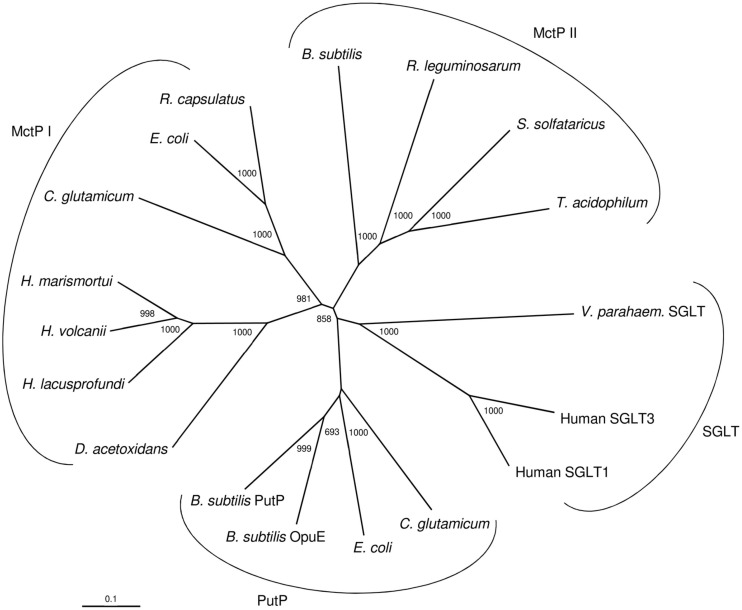
Phylogenetic relationship of the acetate transporter ActP from *H. volcanii* and related members of the sodium: solute symporter family from eukarya, bacteria and archaea. The tree is based upon a multiple sequence alignment that was generated with ClustalX. Numbers at the nodes are bootstrapping values according to neighbor joining. UniProt accession numbers are as follows: MctP I: *Haloferax volcanii*, (HVO_A0154) D4GQI3; *Haloarcula marismortui*, Q5UXS5; *Halorubrum lacusprofundi*, B9LP25; *Desulfuromonas acetoxidans*, Q1JVX2; *E. coli*, P32705; *Rhodobacter capsulatus*, D5APM1; *Corynebacterium glutamicum*, Q8NS49; MctP II: *Rhizobium leguminosarum*, Q1M7A2; *Sulfolobus solfataricus*, (SSO2113) Q7LX83; *Thermoplasma acidophilum*, (Ta0300) Q9HLC8; *Bacillus subtilis*, (BSU19580) O34745; Sodium: proline symporter (PutP): *Corynebacterium glutamicum*, Q79VH1; *E. coli*, P07117; *Bacillus subtilis* (OpuE) O06493; *Bacillus subtilis*, (PutP) P94392; Sodium: sugar symporter (SGLT): Human, (SGLT3) Q9NY91; Human (SGLT1) P13866; *Vibrio parahaemolyticus* (SGLT) P96169.

A phylogenetic relationship of ActP from *H. volcanii* and selected characterized and putative homologs - all of which being members of the SSF family - is shown in Figure 4. The homologs form four distinct clusters, two monocarboxylate transporter cluster, MctP I and MctP II, the sodium: proline (PutP) and sodium: glucose/galactose symporter (SGLT) cluster. The MctP I cluster includes ActP from *H. volcanii* and the putative homologs of the haloarchaea *Haloarcula marismortui* and *Halorubrum lacusprofundi*, and of the acetate utilizing bacterium *Desulfuromonas acetoxidans*. The cluster also includes the characterized monocarboxylate transporter ActP of *E. coli*, MctC of *C. glutamicum* and ActP of *Rhodobacter capsulatus* (Borghese and Zannoni, 2010). The acetate transporter of *H. volcanii* represents the first characterized carrier of the SSF family in the archaeal domain. The MctP II cluster includes the characterized transporter MctP of *Rhizobium leguminosarum* (Hosie et al., 2002). This cluster also includes closely related putative homologs from the bacterium *Bacillus subtilis* and the thermoacidophilic archaea *Sulfolobus solfataricus* and *Thermoplasma acidophilum*. The transport function of these homologs has to be analyzed. The PutP cluster includes characterized sodium-dependent proline uptake systems of bacteria, e.g., of *Bacillus subtilis* (Moses et al., 2012) and *E. coli* (Jung et al., 2012), and the SGLT cluster includes sodium-dependent symporters for glucose in human and for galactose in *Vibrio parahaemolyticus*. This separate clustering of SSF uptake systems according to their distinct transport function has been reported previously (Jung, 2002; Jolkver et al., 2009). It should be noted that the acetate transporter from methanoarchaeal *Methanosarcina* species belongs to a different protein family, the AceTr family (Ribas et al., 2019).

### Acetate Activation in *H. volcanii* Is Mediated by Different Types of AMP-forming Acetyl-CoA Synthetases

In genomic vicinity to the *ssf* gene the gene *acs7* is located that encodes a putative AMP-forming acetyl-CoA synthetase (ACS7). *Acs7* is most likely part of the acetate-inducible operon and thus a candidate to activate acetate to acetyl-CoA during growth on acetate. However, the genome of *H. volcanii* contains eight additional ACS-like paralogs (Table 1), which might also be involved in acetate activation, e.g., ACS1 that shows a high sequence identity (87%) to ACS7. In the following we report the identification of four ACS enzymes that are functionally involved in the activation of acetate in *H. volcanii*.

**TABLE 1:** Genes encoding putative AMP-forming acetyl-CoA synthetases in *H. volcanii*.

	*acs1*	*acs2*	*acs3*	*acs4*	*acs5*	*acs6*	*acs7*	*acs8*	*acs9*
*acs1* HVO_0894	100	16	20	15	35	12	87	63	14
*acs2* HVO_0896		100	25	32	15	17	15	16	69
*acs3* HVO_1236			100	22	22	16	21	20	26
*acs4* HVO_1374				100	16	16	16	16	33
*acs5* HVO_1585					100	12	34	33	16
*acs6* HVO_1917						100	13	13	17
*acs7* HVO_A0156							100	64	14
*acs8* HVO_A0158								100	15
*acs9* HVO_A0551									100

*Sequence identities are given in %.*

After homologous expression of *acs7* the recombinant ACS7 was purified and characterized as a monomeric 79.3 kDa protein. ACS7 catalyzed the CoA- and ATP-dependent activation of acetate to acetyl-CoA with an apparent Vmax value of 26.1 U/mg. The apparent K_m_ values (in mM) for acetate, ATP and CoA were 0.84, 0.59 and 0.23, respectively (Table 2). The enzyme also catalyzed the reverse reaction, i.e., the PP_i_- and AMP-dependent conversion of acetyl-CoA to acetate with an apparent Vmax value of 6.2 U/mg and apparent Km values (in mM) for acetyl-CoA, AMP and PP_i_ of 0.15, 0.09 and 0.37, respectively (Table 2). Highest activity was found at a pH of 6.75 and at KCl concentrations between 3 and 4.5 M. Activity was dependent on MgCl_2_ with an optimal concentration of 2.5 mM.

**TABLE 2:** Molecular and kinetic properties of recombinant ACSs from *H. volcanii*.

	substrate	ACS7	ACS2	ACS1
Subunit (kDa)^1^		77	69	82
Calculated subunit (kDa)		74.0	59.8	71.2
Holoenzyme (kDa)^2^		79.3	139	150
Oligomerization		α	α_2_	α_2_
**Acetate activation:**				
K_m_ value (mM)	acetate	0.84	1.5	0.09
	ATP	0.59	0.43	0.48
	CoA	0.23	2.21	0.73
V_max_ value (U/mg)		26.1	1.5	0.4
**Acetate formation:**				
K_m_ value (mM)	acetyl-CoA	0.15	ND^3^	0.57
	AMP	0.09	ND^3^	0.15
	PP_i_	0.37	ND^3^	0.41
V_max_ value (U/mg)		6.2	ND^3^	0.25

*ND = not detectable.*

*^1^Analyzed by SDS-PAGE.*

*^2^Analyzed by Superdex HiLoad 200.*

*^3^measured with ACS purified from H. volcanii.*

To test the involvement of ACS7 in acetate activation *in vivo*, an *acs7* deletion mutant was generated. Growth of Δ*acs7* mutant on acetate was not affected suggesting that ACS7 might be functionally replaced by one or several ACS paralogs. To identify ACS paralogs involved in acetate activation we purified ACS activity from acetate-grown *H. volcanii* cells using four chromatographic steps yielding a pure protein of 139 kDa. SDS-PAGE analysis revealed a single band at about 69 kDa (Supplementary Figure S2) indicating a homodimeric structure of this ACS. The enzyme had a specific activity of 3.5 U/mg and showed apparent K_m_ values of 2.3 mM for acetate, 1.3 mM for CoA and 0.2 mM for ATP. The enzyme did not show measurable activity in the reverse direction, i.e., in acetate formation from acetyl-CoA. By MALDI-TOF analysis of the 69 kDa subunit, *acs2* was identified as the gene encoding ACS2 (Table 1). The *acs2* gene was overexpressed in *H. volcanii* followed by purification on Ni-NTA and size exclusion chromatography. The molecular and kinetic properties of the recombinant ACS2 were similar to those of the enzyme purified from acetate-grown cells (Table 2). ACS2 was strictly dependent on Mg^+^-ions with a K_m_ value of 0.37 mM. Maximal ACS activity was measured at a pH value of about 8.0 and at potassium chloride concentrations higher than 2 M.

**FIGURE 5:**
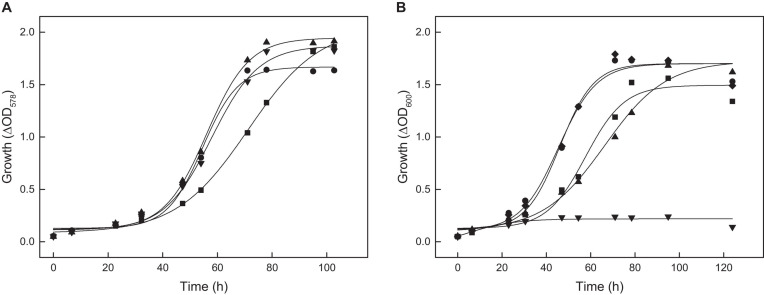
Growth of deletion mutants of different *acs* genes of *H. volcanii* on 40 mM acetate. **(A)** Growth of the Δ*acs2*Δ*acs7* double mutant (■) and the complemented double mutant with *acs2* (▼) and with *acs7* (▲) compared to the wild type (•). **(B)** Growth of the Δ*acs9* mutant (■), Δ*acs9*Δ*acs2*Δ*acs7* triple mutant (▲), the quadruple Δ*acs9*Δ*acs2*Δ*acs7*Δ*acs1* mutant (▼) and the quadruple mutant complemented with *acs9* compared to the wild type (•).

To test the function of ACS2 *in vivo*, an *acs2* deletion mutant, Δ*acs2*, was generated. As shown for the Δ*acs7* mutant growth of the Δ*acs2* mutant on acetate was not affected, but the double-mutant Δ*acs2*Δ*acs7* showed a reduced growth rate by 32% as compared to the wild type (Figure 5A). Wild type growth could be recovered by complementation *in trans* with either *acs2* or *acs7*. The data indicate that ACS2 and ACS7 are functionally involved in the activation of acetate to acetyl-CoA in *H. volcanii*, but can be replaced by other ACS-paralogs that contribute to acetate activation *in vivo.* In search for likely candidates we characterized the most related ACS paralogs, i.e., ACS1 showing 87% sequence identity to ACS7 and ACS9 showing 69% identity to ACS2 (Table 1). Recombinant ACS1 was characterized as 150 kDa homodimeric protein that catalyzed the activation of acetate to acetyl-CoA and also the reverse reaction, the conversion of acetyl-CoA to acetate. Kinetic constant are given in (Table 2). Highest ACS1 activity was measured at 3.5 M KCl and at a pH of 7.0. Recombinant ACS9 was characterized as homodimeric 105 kDa protein composed of 59 kDa subunits. However, a catalytic activity analyzed in direction of acetate activation could not be detected.

The functional role of ACS1 and ACS9 during growth on acetate was analyzed by appropriate deletion mutants. Growth of Δ*acs1* mutant on acetate was not significantly affected; whereas the growth rate of Δ*acs9* mutant was reduced by 20% (Figure 5B). A triple mutant Δ*acs2*Δ*acs7Δacs9* showed a 35% reduced growth rate on acetate. Finally, we prepared the quadruple mutant Δ*acs2*Δ*acs7Δacs9Δacs1*, which completely lost the ability to grow on acetate. Wild type growth of all these mutants could be recovered by complementation with *acs9 in trans* as shown for the quadruple mutant in Figure 5B.

Together, the knockout mutant experiments indicate that ACS7, ACS2, ACS1 and ACS9 contribute to the activation of acetate *in vivo*. The essential role of ACS9 for growth on acetate was obvious from the single deletion mutant, whereas the functional involvement of ACS7, ACS2, and ACS1 could only be demonstrated by double, triple and quadruple mutants. The data exclude an essential role of the other remaining five putative ACS paralogs in *H. volcanii* (Table 1) during growth on acetate.

So far, an ACS from the haloarchaeon *H. marismortui* has been biochemically characterized (Bräsen and Schönheit, 2005). It is a monomeric 72 kDa protein showing high specificity for acetate using only propionate at low activity. It showed the highest sequence identity (73%) to ACS7 from *H. volcanii*. However, *H. marismortui* contained several ACS paralogs, which have not yet been characterized. It can be speculated that more than one ACS in *H. marismortui* contribute to the activation of acetate as found in *H. volcanii*. Further, several ACS proteins have been characterized from other archaea, e.g., from *Methanothermobacter* species *M. thermoautotrophicus* and *M. marburgensis*, from the *Methanosaeta* species *M. concilii* and *M. thermophila* and *Archaeoglobus fulgidus*. These ACS are monomeric and dimeric enzymes of about 70 kDa subunits showing high specificity for acetate and utilizing propionate to some extent (Oberlies et al., 1980; Jetten et al., 1989; Ingram-Smith and Smith, 2007; Berger et al., 2012). The ACS of the hyperthermophilic archaeon *Pyrobaculum aerophilum* is an extreme thermoactive (T_opt_ > 97°C) homooctameric protein of 75 kDa subunits, that showed a high affinity for acetate but also accepts formate, propionate, butyrate and isobutyrate at significant rates (Bräsen et al., 2005). An octameric ACS of 75 kDa subunits has also been reported for the hyperthermophilic archaeon *Ignicoccus hospitalis* (Mayer et al., 2012). A high oligomerization state has been implicated in stabilization of proteins at high temperatures (Sterner and Liebl, 2001). ACS from bacteria have been characterized mostly as either monomeric or homodimeric enzymes composed of subunits of about 70 kDa (Bräsen et al., 2005).

### Phylogenetic Affiliation of ACS Homologs From *H. volcanii*

The nine ACS paralogs from *H. volcanii* (Table 1) belong to the acyl-CoA synthetase family which is part of the superfamily of acyladenylate/thioester-forming enzymes (Babbitt et al., 1992). Acyl-CoA synthetases activate fatty acids to acyl-CoA thioesters via a common mechanism involving two partial reactions (Starai and Escalante-Semerena, 2004). In the first ATP-dependent reaction fatty acids are converted to an acyl-AMP intermediate and PP_i_; in a second - CoA-dependent reaction - acyl-CoA and AMP are formed. All acyl-CoA synthetases contain a conserved lysine that is essential in the formation of the acyl-AMP intermediate. This catalytic lysine is also conserved in eight of the nine paralogs from *H. volcanii*, with exception of ACS6, which is thus considered to be catalytic inactive.

**FIGURE 6:**
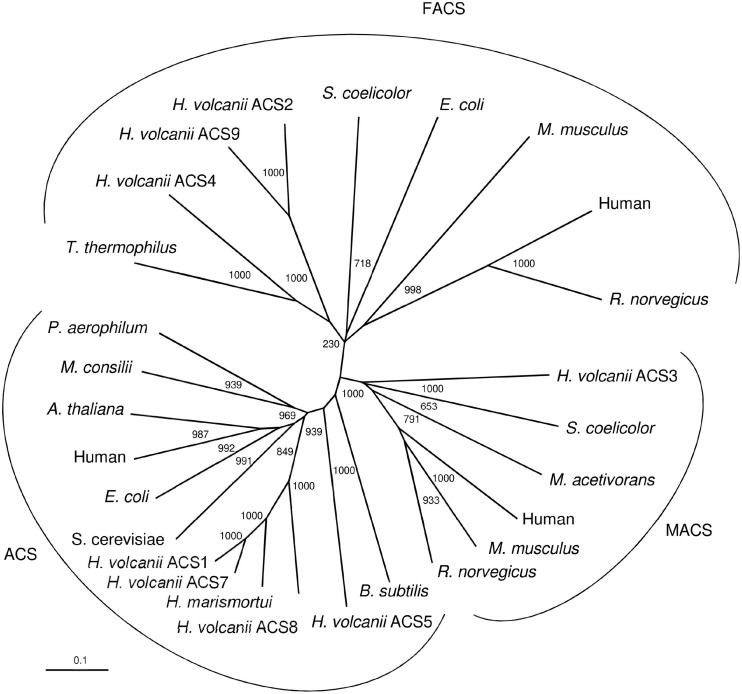
Phylogenetic relationship of ACS proteins from *H. volcanii* and characterized and putative members of the three ACS families, ACS, MACS (medium chain acyl-CoA synthetases) and FACS (long chain acyl-CoA synthetases). UniProt accession numbers are as follows: ACS: *Bacillus subtilis*, P39062; *Haloferax volcanii*, ACS5; *Haloferax volcanii*, ACS8; *Haloarcula marismortui*, Q5UXS3; *Haloferax volcanii*, ACS7; *Haloferax volcanii*, ACS1; *Saccharomyces cerevisiae*, P52910; *Escherichia coli*, P27550; Human, Q9NR19; *Arabidopsis thaliana*, B9DGD6; *Methanosaeta concilii*, P27095; *Pyrobaculum aerophilum*, O93730. MACS: *Haloferax volcanii*, ACS3; *Streptomyces coelicolor*, NP_630298; *Methanosarcina acetivorans*, Q8TLW1; Human, Q08AH1; *Mus musculus*, Q91VA0; *Rattus norvegicus*, Q7TN78. FACS: *Thermus thermophilus*, PDB entry 1V25; *Haloferax volcanii*, ACS4; *Haloferax volcanii*, ACS9; *Haloferax volcanii*, ACS2; *Streptomyces coelicolor*, QFI46026; *Escherichia coli*, P69451; *Mus musculus*, Q99PU5; Human, P33121; *Rattus norvegicus*, O88813.

ACS enzymes can be classified according to their specificity to activate fatty acids of different chain lengths as acetyl-CoA synthetases (ACS) that activate predominantly acetate (C2), medium chain acyl-CoA synthetases (MACS) and long chain acyl-CoA synthetases (FACS) which activate fatty acids with C4 to C12 and C12 to C22, respectively (Black and DiRusso, 2003; Shah et al., 2009). A phylogenetic relationship between the *Haloferax* ACS proteins and characterized and putative members of the three ACS families, ACS, MACS and FACS is shown in Figure 6. In accordance with previous analyses, ACS, MACS and FACS form distinct phylogenetic cluster (Fujino et al., 2001; Oba et al., 2005). The ACS cluster includes selected characterized and putative ACS from eukarya, bacteria and archaea, including the characterized ACS from *H. marismortui* and ACS1 and ACS7 from *H. volcanii*. Also, the putative ACS5 and ACS8 from *H. volcanii* are members of this cluster although these enzymes are not involved in acetate activation. Detailed phylogenetic analyses of ACS from archaea, bacteria and eukarya have previously been reported (Bräsen and Schönheit, 2005; Ingram-Smith et al., 2006). The MACS cluster includes characterized members of eukarya, bacteria and of *M. acetivorans*. The putative ACS3 from *H. volcanii* is part of the MACS cluster suggesting a catalytic function in activating medium chain fatty acids. The FACS cluster includes characterized members of eukarya, bacteria, the characterized ACS2 and ACS9 and the putative ACS4 from *H. volcanii*. The phylogenetic attribution of ACS2 and ACS9 - shown to be functional involved in acetate activation - in the FACS cluster was unexpected, since FACS proteins predominantly activate fatty acids with high chain length C12-C22. The specificity of ACS2 and ACS9 toward long chain fatty acids could not be tested due to the precipitation of fatty acids > C10 in the presence of high salt concentration required in the assay system.

Finally, we have shown that four different ACS enzymes are involved in acetate activation in *H. volcanii* whereby ACS7 is part of an operon together with the acetate transporter ActP. It is interesting to note that a similar operon structure, comprising homologous proteins is also present in *H. marismortui* and *H. lacusprofundi*, suggesting a role of putative transporter and of ACS in uptake and activation of acetate. Further, an operon structure of ACS and SSF is also present in the bacteria *E. coli* and *Rhodobacter capsulatus* (Supplementary Figure S3). For *E. coli* an acetate specific induction of the operon has been indicated (Gimenez et al., 2003). Also, *C. glutamicum* contains an homologous acetate transporter, but an ACS homolog is missing in the cluster, since the organism activates acetate to acetyl-CoA via acetate kinase and phosphotransacetylase (Gerstmeir et al., 2003).

### Acetyl-CoA Conversion to Phosphoenolpyruvate Involves Isocitrate Lyase, Two Malic Enzymes and Phosphoenolpyruvate Synthetase

**FIGURE 7:**
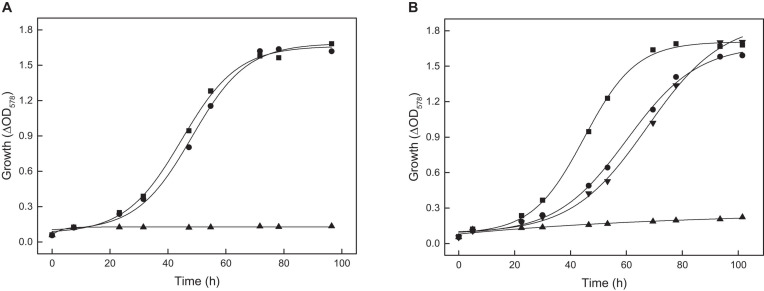
Growth of the *aceA*, *tme* and *mdh* deletion mutants of *H. volcanii* on 40 mM acetate. The genes *aceA*, *tme*, and *mdh* encode isocitrate lyase, Mae1 and Mae2, respectively. **(A)** Growth of the Δ*aceA* mutant (▲) and the complemented mutant with *aceA* (•) compared to the wild type (■). **(B)** Growth of the *tme* and *mdh* double deletion mutant (▲) and the complemented double mutant with *tme* (▼) or *mdh* (•) compared to the wild type (■).

In the anabolism, acetyl-CoA serves as substrate for synthesis of malate and phosphoenolpyruvate (PEP) as part of anaplerosis and gluconeogenesis. Previous studies indicate that in *H. volcanii* acetyl-CoA is converted to malate via reactions of the glyoxylate pathway. The key enzymes of this pathway, isocitrate lyase and malate synthase, have been characterized (Serrano et al., 1998; Serrano and Bonete, 2001; Falb et al., 2008). To prove the functional involvement of isocitrate lyase, encoded by the *aceA* gene, a deletion mutant was generated. The Δ*aceA* mutant did not grow on acetate (Figure 7A), and wild type growth was recovered upon *in trans* complementation with *aceA* indicating an essential function of the glyoxylate pathway in *H. volcanii* during growth on acetate. The data exclude the operation of the methylaspartate cycle, the alternative anaplerotic pathway reported for *Haloarcula marismortui* (Borjian et al., 2016).

In most aerobic bacteria growing on acetate, malate formed in glyoxylate cycle is converted to PEP via PEP carboxykinase (PEPCK) (Sauer and Eikmanns, 2005). In the genome of *H. volcanii* a gene encoding PEPCK is absent. Instead, two genes, *tme* and *mdh*, that encode putative malic enzymes and a gene, *ppsA*, encoding a putative PEP synthetase are annotated. In the following we describe the characterization of the two malic enzymes and PEP synthetase and the functional involvement of the enzymes during growth on acetate.

The putative malic enzymes encoded by *tme* and *mdh* of *H. volcanii* were designated Mae1 and Mae2, respectively. Both proteins show an amino acid sequence identity of 83% to each other and a calculated molecular weight of 81.4 kDa. *Tme* and *mdh* were overexpressed in *H. volcanii* and the recombinant proteins were purified by Ni-NTA affinity chromatography and size exclusion chromatography. Mae1 showed on SDS-PAGE a single band at 91.5 kDa; by gel filtration a molecular mass of 612 kDa was determined indicating a homooctameric structure of the enzyme. Mae1 catalyzed the NADP^+^-dependent decarboxylation of malate to pyruvate with an apparent V_max_ value of 3.2 U/mg. The apparent Km values for malate and NADP^+^ were 0.65 mM and 0.06 mM, respectively. NADP^+^ could not be replaced by NAD^+^ as electron acceptor. Highest activity was measured at KCl concentrations higher than 2 M, at MnCl_2_ concentrations higher than 2 mM and a pH of 5.75. The enzyme also catalyzed the reverse reaction, the NADPH dependent carboxylation of pyruvate to malate with a Vmax value of 11.2 U/mg and apparent Km values of 0.1 mM for NADPH, 3.95 mM for pyruvate and 5.5 mM for NaHCO_3_. NADH was not used an electron donor. Mae2 was characterized as 530 kDa protein composed of 81 kDa subunits. Mae2 catalyzed the NAD^+^-dependent decarboxylation of malate to pyruvate following Michaelis-Menten kinetics with a Vmax value of 1.5 U/mg and a Km value of 107 mM for malate. The Mae2 also uses NADP^+^ as cofactor. The apparent Km values for NAD^+^ and NADP^+^, measured at 10 mM malate were 0.15 and 0.1 mM, respectively. The corresponding Vmax values were 0.17 and 0.05 U/mg indicating a preference of Mae2 for NAD^+^. Mae2 also catalyzed the NADH-dependent carboxylation of pyruvate with a Vmax of 0.113 U/mg and a Km value for pyruvate of 112 mM.

To prove the functional involvement of Mae1 and Mae2 in acetate metabolism of *H. volcanii* single deletion mutants were generated. Growth of either Δ*tme* or Δ*mdh* mutant on acetate was not affected (not shown) suggesting that Mae1 and Mae2 functionally replace each other. In accordance the double mutant Δ*tme*Δ*mdh* completely lost the ability to grow on acetate. Growth could be recovered upon complementation *in trans* with either *tme* or with *mdh* (Figure 7B). The data indicate that both malic enzymes together are essential for growth of *H. volcanii* on acetate.

Malic enzymes have been characterized from bacteria and eukarya, and from few archaea. Bacterial malic enzymes are either composed of 50–60 kDa subunits or of larger, 80–90 kDa, subunits; the latter contain a C-terminal extension showing high similarity to phosphotransacetylase (PTA domain). In bacteria, e.g., in *E. coli*, the PTA domain has been shown to have a function in regulation of malic enzyme activity (Bologna et al., 2007). Like the large bacterial enzymes, the malic enzymes Mae1 and Mae2 from *H. volcanii* are composed of about 80 kDa subunits containing a C-terminal PTA domain. In contrast, malic enzymes that have been characterized from other archaea, from the hyperthermophiles *Sulfolobus solfataricus* and *Thermococcus kodakarensis*, are composed of 50 kDa subunits that do not contain a PTA domain. These archaeal enzymes are homodimers and thus differ from Mae1 from *H. volcanii*, which constitutes a 612 kDa homooctameric protein; this unusual oligomerization as an octamer has also been reported for MaeB from *E. coli* which is composed of large 83 kDa subunits containing a PTA domain (Bologna et al., 2007).

Mae1 and Mae2 from *H. volcanii* showed highest sequence identity (about 75%) to putative enzymes from the haloarchaea *Haloarcula marismortui*, *Halorubrum lacusprofundi*, *Haloterrigena turkmenica* and *Halobacterium salinarum*. All these haloarchaeal malic enzymes are composed of larger subunits containing a C-terminal PTA domain. High sequence identity (about 45%) were also found with those bacterial malic enzymes that are composed of large, PTA domain containing, subunits; these include e.g., MaeB from *E. coli.* Less sequence identity (about 20%) to Mae1 and Mae2 of *H. volcanii* were found with malic enzymes from other archaea and from bacteria that do not contain a PTA domain and about 10% sequence identity were found with malic enzymes from eukarya.

**FIGURE 8:**
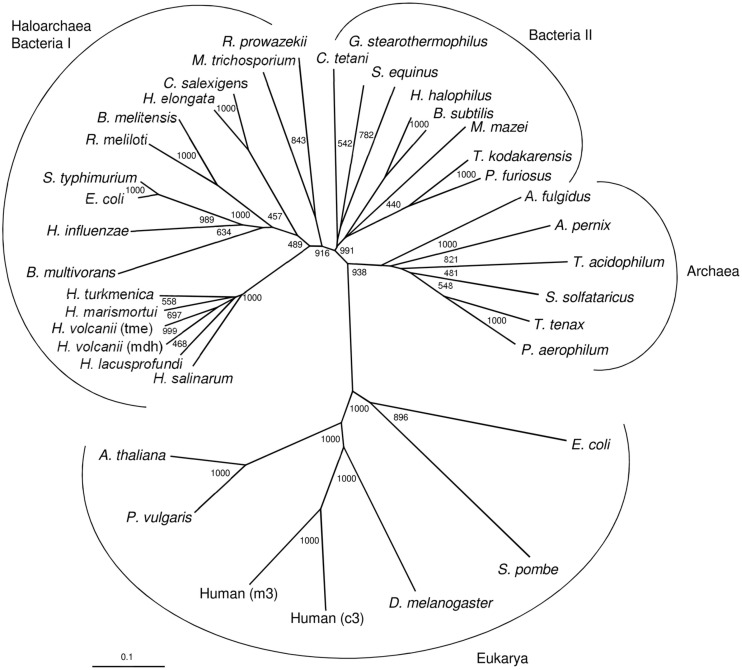
Phylogenetic relationship of the two malic enzymes from *H. volcanii* and malic enzymes from bacteria, other archaea and eukarya. The tree is based upon a multiple sequence alignment that was generated with ClutalX. Numbers at the nodes are bootstrapping values according to neighbor joining. UniProt accession numbers are as follows: Haloarchaea/Bacteria I: *Haloferax volcanii*, HVO_2158; *Haloferax volcanii* (tme) HVO_2436; *Haloarcula marismortui*, rrnAC1754; *Halorubrum lacusprofundi*, Hlac_1525; *Haloterrigena turkmenica*, Htur_2471; *Halobacterium salinarum*, Q9HPI2; *Escherichia coli*, P76558; *Salmonella typhimurium*, Q9ZFV8; *Haemophilus influenzae*, P43837; *Rhizobium meliloti*, O30807; *Halomonas elongata*, E1V8J1; *Chromohalobacter salexigens*, Q1QZZ1; *Brucella melitensis*, Q8YH37; *Rickettsia prowazekii*, Q9ZDF6; *Burkholderia multivorans*, B9B9B2; *Methylosinus trichosporium*, A0A2D2D039. Bacteria II: *Halobacillus halophilus*, I0JPP2; *Geobacillus stearothermophilus*, P16468; *Bacillus subtilis*, O34962; *Streptococcus equinus*, Q59826; *Thermococcus kodakarensis*, Q5JGC7; *Methanosarchina mazei*, Q8PTT0; *Pyrococcus furiosus*, Q8U225; *Clostridium tetani*, A0A4Q0V1F5. Archaea: *Thermoproteus tenax*, G4RKP8; *Sulfolobus solfataricus*, Q97UX2; *Archaeoglobus fulgidus*, O28547; *Aeropyrum pernix*, Q9YF49; *Thermoplasma acidophilum*, Q9HKY7; *Pyrobaculum aerophilum*, Q8ZWP3. Eukarya: *Drosophila melanogaster*, Q9U1J0; Human, (c3) P48163; Human, (m3) Q16798; *Phaseolus vulgaris*, P12628; *Shizosaccharomyces pombe*, P40375; *Arabidopsis thaliana*; O82191*; Escherichia coli*, (b1479) P26616.

A phylogenetic tree including selected characterized and putative sequences of malic enzymes from archaea, bacteria and eukarya is shown in Figure 8. As previously reported (Fukuda et al., 2005), the sequences from prokaryotes, bacteria and archaea, are clearly separated from the eukaryal sequences forming two distinct clusters. Within the prokaryotic clusters three distinct clades, one comprising the haloarchaeal enzymes and those homologs from bacteria that contain large subunits containing a PTA domain. A second clade is composed of malic enzymes from other bacteria that all do not contain a PTA domain (small subunits). This cluster also includes few archaeal malic enzymes from *M. mazei*, *Thermococcus kodakarensis* and *Pyrococcus furiosus*. The third clade comprises other archaeal homologs that are all composed of small subunits. Thus, the tree topology suggests that the haloarchaeal malic enzymes followed a separate evolutionary line distinct from those of other archaea.

**FIGURE 9:**
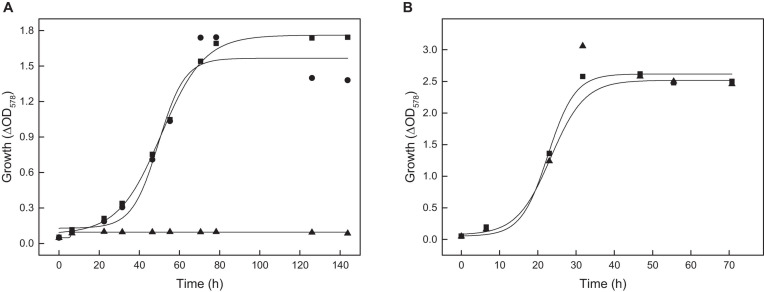
Growth of the *ppsA* deletion mutant of *H. volcanii* on acetate and glucose. **(A)** Growth of the Δ*ppsA* mutant on 40 mM acetate (▲) and the complemented mutant with *ppsA* (•) compared to the wild type (■). **(B)** Growth of the Δ*ppsA* mutant on 15 mM glucose (▲) compared to the wild type (■).

The gene *ppsA* encodes a putative phosphoenolpyruvate synthetase (PPS). *PpsA* was overexpressed in *H. volcanii* and the recombinant enzyme was purified by Ni-NTA affinity chromatography yielding pure protein. SDS-PAGE revealed a single band at 87.9 kDa. The enzyme catalyzed the ATP-dependent conversion of pyruvate to PEP, AMP and phosphate with a Vmax value of 0.157 U/mg and Km values of 0.27 mM for pyruvate and 0.36 mM for ATP; a catalytic activity measured in the reverse reaction, the AMP- and phosphate-dependent pyruvate formation from PEP, could not be detected. A *ppsA* deletion mutant Δ*ppsA* was generated and analyzed under gluconeogenetic and glycolytic growth conditions. The mutant did not grow on acetate and complementation with *ppsA in trans* restored growth (Figure 9A); the mutant did also not grow on pyruvate (not shown). In contrast, growth of *H. volcanii* on glucose was not affected by the *ppsA* deletion (Figure 9B). These results clearly indicate that PPS from *H. volcanii* is exclusively involved in gluconeogenesis and exclude a function in glycolysis.

So far, PEP synthetases have been characterized from several archaea, e.g., *Haloferax mediterranei*, *Thermoproteus tenax*, *Staphylothermus marinus*, *Methanobacterium thermoautotrophicum*, *Pyrococcus furiosus* and *Sulfolobus solfataricus* (Eyzaguirre et al., 1982; Cicicopol et al., 1999; Hutchins et al., 2001; Tjaden et al., 2006; Chen et al., 2019; Haferkamp et al., 2019). All archaeal PPSs show similar subunit sizes (75-90 kDa) that have also been reported for PPSs from bacteria and eukarya. The PPSs from archaea, bacteria and eukarya are members of the PEP-utilizing enzyme family (Chen et al., 2019).

PPS from *H. volcanii* has been shown to exclusively operate in the gluconeogenetic direction. A similar role of PPS in gluconeogenesis has also been reported for *Haloferax mediterranei*; the respective Δ*pps* mutant did not grow on pyruvate whereas growth on the glycolytic substrate glycerol was not affected (Chen et al., 2019). As shown for the *H. volcanii* PPS, the enzymes from *H. mediterranei*, *T. tenax, P. furiosus* and *S. solfataricus* catalyze - almost exclusively - the unidirectional conversion of pyruvate to PEP, which is in accordance with a physiological role of these archaeal PPS in gluconeogenesis. However, in contrast, PPSs from the *Thermococcales*, *P. furiosus* and *T. kodakarensis*, have been proposed to have an additional glycolytic function. A Δ*pps* mutant from *T. kodakarensis* did not grow on malto-oligosaccharides indicating a role in sugar degradation (Imanaka et al., 2006). So far, the glycolytic role of PPS in *Thermococcales*, which employ an unusual Embden-Meyerhof pathway utilizing ADP-dependent kinases generating AMP, is still a matter of debate (Hutchins et al., 2001; Sakuraba et al., 2001; Imanaka et al., 2006).

## Conclusion

**FIGURE 10:**
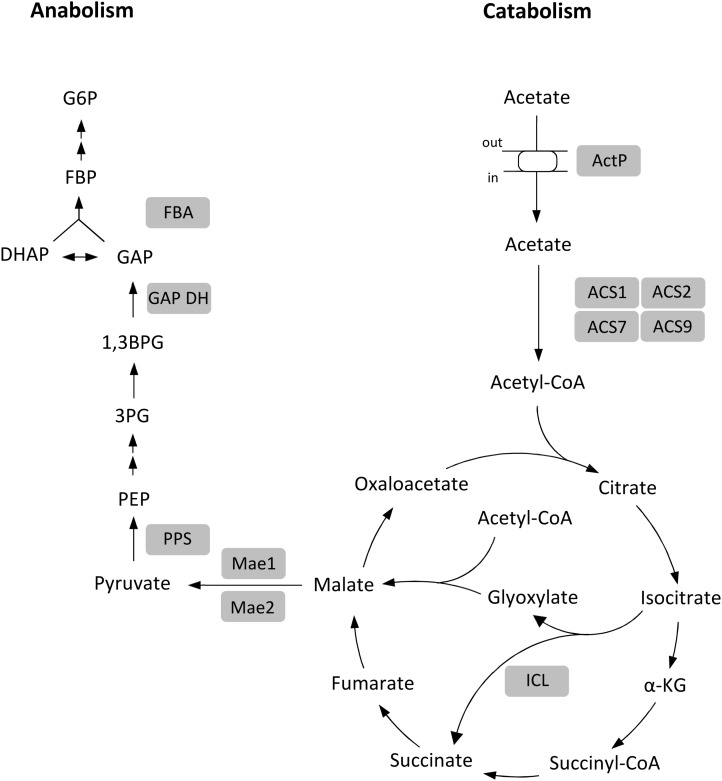
Proposed metabolism of acetate in *H. volcanii*. Enzymes shown to be functionally involved in acetate metabolism are highlighted by gray background. α-KG, α-ketoglutarate; PEP, phosphoenolpyruvate; 3PG, 3-phosphoglycerate; 1,3BPG, 1,3-bisphosphoglycerate; GAP, glyceraldehyde-3-phosphate; DHAP, dihydroxyacetone phosphate; FBP, fructose-1,6-bisphosphate; G6P, glucose-6-phosphate; ActP, acetate permease; ACS1, ACS2, ACS7 and ACS9, acetyl-CoA synthetases; ICL, isocitrate lyase; Mae1 and 2, malic enzymes; PPS, PEP synthetase; GAPDH, GAP dehydrogenase; FBA, FBP aldolase.

In this study we report a comprehensive analysis of acetate metabolism in archaea, in the haloarchaeon *Haloferax volcanii*. The genes and enzymes which were involved in the uptake and activation of acetate and in gluconeogenesis have been identified and characterized. Conclusive evidence for their functional involvement was obtained by growth studies with respective knock-out mutants. The data are summarized in Figure 10. The uptake of acetate involves a transporter of the SSF family; thus, the *H. volcanii* transporter represents the first characterized archaeal member of this family. The activation of acetate to acetyl-CoA involves four paralogous ACS enzymes that belong to two different groups of ACS proteins. Further, we showed that malate formation from acetyl-CoA proceeds via glyoxylate pathway and we identified the enzymes catalyzing the synthesis of PEP from malate involving two malic enzymes and PPS. The complete loss of growth on acetate of the knock-out mutants of the respective encoding genes excludes alternative anaplerotic or gluconeogenetic pathways. Finally, we include in Figure 10 the anabolic glyceraldehyde-3-phosphate dehydrogenase (Tästensen and Schönheit, 2018) and fructose-1,6-bisphosphate aldolase (Pickl et al., 2012) since these enzymes have been shown to be essential for gluconeogenesis during growth on acetate.

## Data Availability Statement

The original contributions presented in the study are included in the article/Supplementary Material, further inquiries can be directed to the corresponding author/s.

## Author Contributions

TK, UJ, and PS designed the experiments and wrote the manuscript. MO characterized Mae2 and performed few growth experiments. TK performed all other experiments. All authors analzyed the data. All authors contributed to the article and approved the submitted version.

## Conflict of Interest

The authors declare that the research was conducted in the absence of any commercial or financial relationships that could be construed as a potential conflict of interest.
