# Supplementary material for: Acetate Metabolism in Archaea: Characterization of an Acetate Transporter and of Enzymes Involved in Acetate Activation and Gluconeogenesis in Haloferax volcanii
Source: Front Microbiol. 2020 Dec 4;11:604926. doi: 10.3389/fmicb.2020.604926 (PMC7746861; doi:10.3389/fmicb.2020.604926)

Supplementary data

Acetate metabolism in archaea: Characterization of an acetate transporter and of enzymes involved in acetate activation and gluconeogenesis in *Haloferax volcanii*

Tom Kuprat[1], Ulrike Johnsen[1], Marius Ortjohann[1] and Peter Schönheit[1*]

[1]Institut für Allgemeine Mikrobiologie, Christian-Albrechts-Universität, Kiel, Germany

Table of contents

Supplemental Figure S1 Growth of *H. volcanii* on acetate. Cells were grown at 42°C on 40 mM acetate (•); consumption of acetate (▪) was monitored over time.

Supplemental Figure S2 SDS-PAGE of purified ACS2 from acetate-grown cells of *H. volcanii*.

Supplemental Figure S3 Genomic view of the acetate operon identified in the haloarchaea *Haloferax volcanii*, *Haloarcula marismortui* and *Halorubrum lacusprofundi* compared to homologous regions found in the bacteria *E. coli* , *R. capsulatus* SB1003 and *C. glutamicum*.

Supplemental Figure S1 Growth of *H. volcanii* on acetate. Cells were grown at 42°C on 40 mM acetate (●); consumption of acetate (■) was monitored over time.

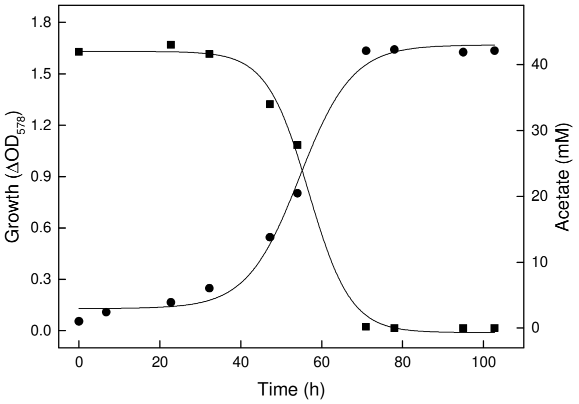

Supplemental Figure S2 SDS-PAGE of purified ACS2 from acetate-grown cells of *H. volcanii*.

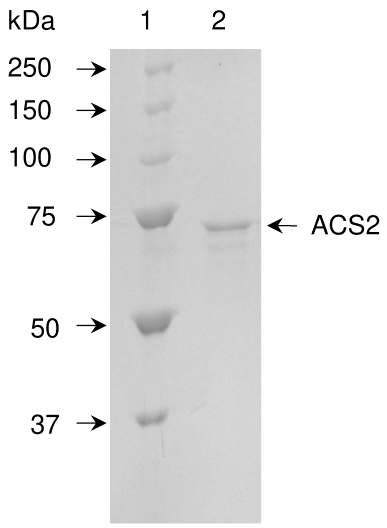

Supplemental Figure S3 Genomic view of the acetate operon identified in the haloarchaea *Haloferax volcanii*, *Haloarcula marismortui* and *Halorubrum lacusprofundi* compared to homologous regions found in the bacteria *E. coli* , *R. capsulatus* SB1003 and *C. glutamicum.*

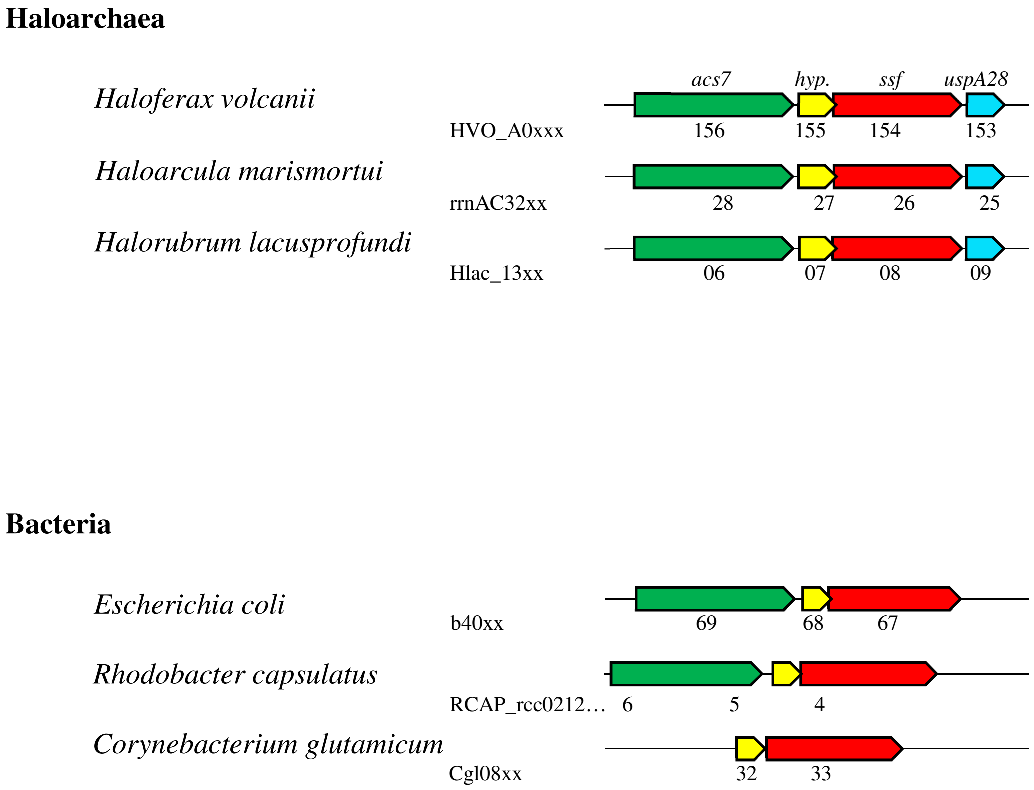

Supplement: Supplementary file 1 [file Data_Sheet_1.PDF]
